# Show Me the Money! Trends in Funding for Health Services Research

**DOI:** 10.1111/1475-6773.13040

**Published:** 2018-09-21

**Authors:** Lisa A. Simpson, Liz Koechlein, Nir Menachemi, Meghan J. Wolfe

**Affiliations:** ^1^ AcademyHealth Washington DC; ^2^ Food and Agriculture Organization United Nations Viale delle Terme di Caracalla Rome Italy; ^3^ Department of Health Policy and Management Richard M. Fairbanks School of Public Health Indiana University Bloomington IN; ^4^ Department of Health Policy and Management Milken Institute School of Public Health The George Washington University Washington DC

**Keywords:** Health workforce, distribution/Incomes/Training, Health policy/Politics/Law/Regulation, Health care organizations and systems

## Abstract

This paper presents longitudinal data representing federal funding for health services research and discusses the observed trends in the larger context of overall funding for research and development in the United States. By putting into context public and private funding trends, the authors examine how these trends effect the supply and demand of the health services research workforce.

Discussion pertaining to the future of the health services research workforce must take into account the context of public and private financial support for work in the field. As noted by other authors in the current special issue, the demand for health services research is diversifying, while estimates of the current workforce point to continued growth. In the present brief report, we present longitudinal data representing federal funding for health services research and place the observed trends in the larger context of overall funding for research and development.

## Trends in Federal Health Services Research Funding

Two data sources were used to summarize trends in federal support for health services research. Total funding for health services research is summarized by AcademyHealth annually using official federal agency budgets and the NIH RePORTER tool for NIH support. The RePORTER system is an electronic tool made available by the National Institutes of Health (NIH) that supports searches for NIH‐funded research projects (both intramural and extramural) from the past 25 years resulting from NIH funding. The reporter system also provides the annual support level for various research, condition, and disease categories based on grants, contracts, and other funding mechanisms used across the National Institutes of Health (NIH). This list contains the category of health services research (NIH Reporter). In addition, trends in the number and distribution of health services research projects funded are tracked by HSRProj, the most comprehensive repository for health services research projects in the United States with data from 360 public and private funding organizations. HSRProj is managed by AcademyHealth and the Cecil G. Sheps Center for Health Services Research at the University of North Carolina, Chapel Hill, on behalf of the US National Library of Medicine. HSRProj contains information on health services research projects spanning decades, including information on ongoing projects before results are available in a published format. The number of projects in HSRProj has increased over time, currently including information on 32,244 research projects, approximately half of which are ongoing or have been completed within the last 5 years. While discussing the methodologies used to categorize health services research projects is outside the scope of this article, we recognize that these data sources may be subject to classification bias in which some health services research‐funded projects are omitted and/or some nonhealth services research projects are erroneously included.

Table 1 displays funding for health services research from US federal agencies during the FY 2010–FY 2017 time frame. In FY 2017,^1^ the National Institutes of Health (NIH) reported spending nearly $1.8 billion on health services research, accounting for 59.5 percent of all federal health services research funding; however, this represents only 5.3 percent of the total budget ($33.1 billion) for NIH in that year. The Affordable Care Act (ACA) established the Patient‐Centered Outcomes Research (PCOR) Trust Fund with a new tax on health plans based on a formula using the number of covered lives and transfers from Treasury. This new fund has provided nearly $1.9 billion since 2010 in support of PCOR, a subset of health services research. However, this new funding source has also been used to supplant base funding for the Agency for HealthCare Research and Quality (AHRQ) since 2013. This budget strategy was a direct result of the intense pressure on discretionary funding created by the Budget Control Act of 2011. However, if the Trust Fund is not reauthorized in 2019, not only would funding for the Patient‐Centered Outcomes Research Institute (PCORI) disappear, AHRQ would lose nearly $100 million, or 22 percent of its budget. Another way to examine trends in AHRQ funding is to examine its budget levels in inflation‐adjusted dollars. In doing so, it reveals that the amount requested by the administration for FY 2019 is $120 million below FY 2010 levels when adjusting for inflation. A final notable comparison is total federal support for health services research compared to national health care expenditures. In FY 2016, the $2.9 billion spent on health services research was less than one‐tenth of one percent of the $3.3 trillion spent on health care overall; and less than a quarter of one percent of the $1.24 trillion spent on Medicare and Medicaid (Centers for Medicare and Medicaid Services 2018).

**Table 1:** Federal Funding for Health Services Research 2010–2017

Agency	FY 2010	FY 2011	FY 2012	FY 2013	FY 2014	FY 2015	FY 2016	FY 2017
Agency for healthcare research and quality	$ 397.00	$ 384.00	$ 405.00	$ 433.00	$ 464.00	$ 465.00	$ 427.60	$ 416.60
Base discretionary		$ 372.00	$ 372.00	$ 369.00	$ 364.00	$ 364.00	$ 334.00	$ 324.00
Prevention and public health fund		$ 12.00	$ 12.00	$ 6.00	$ 7.00			
Patient‐centered outcomes research fund		$ 8.00	$ 24.00	$ 57.50	$ 93.00	$ 101.00	$ 93.60	$ 92.15
CDC: National centers for health statistics	$ 138.70	$ 168.00	$ 168.00	$ 168.00	$ 153.90	$ 155.40	$ 160.40	$ 160.40
Base discretionary	$ 138.70	$ 138.00	$ 138.00	$ 138.00	$ 153.90	$ 155.40	$ 160.40	$ 160.40
Prevention and public health fund		$ 30.00	$ 30.00	$ 30.00				
CDC: Public health research/PHSSR	$ 31.20	$ 21.00	$ –	$ –	$ –	$ –	$ –	$ –
Base discretionary		$ 11.00						
Prevention and public health fund		$ 10.00						
CDC: Prevention research centers	$ 33.70	$ 28.00	$ 28.00	$ 23.40	$ 25.50	$ 25.50	$ 25.50	$ 25.50
Base discretionary		$ 18.00	$ 18.00	$ 8.10	$ 25.50	$ 25.50	$ 25.50	$ 25.50
Prevention and public health fund		$ 10.00	$ 10.00	$ 15.30				
CMS: Research, demonstration & evaluation projects	$ 36.00	$ 35.00	$ 21.20	$ 20.10	$ 20.10	$ 20.10	$ 20.10	$ 20.10
HRSA: Rural health policy development	$ 10.00	$ 9.90	$ 10.00	$ 9.30	$ 9.40	$ 9.40	$ 9.40	$ 9.40
National institutes of health^a^	$ 1,131.00	$ 1,116.00	$ 1,164.00	$ 1,262.00	$ 1,342.00	$ 1,437.00	$ 1,692.00	$ 1,770.00
Veterans health administration	$ 84.00	$ 91.30	$ 90.00	$ 90.30	$ 96.00	$ 91.30	$ 97.80	$ 104.60
PCORI	$ 50.00	$ 120.00	$ 161.60	$ 240.70	$ 425.70	$ 422.50	$ 471.00	$ 466.10
Total (Program level)	$ 1,912.00	$ 1,973.00	$ 1,958.00	$ 2,241.00	$ 2,536.00	$ 2,627.00	$ 2,904.00	$ 2,972.70

a NIH Estimates of Funding for Various Research, Condition, and Disease Categories (RCDC). https://report.nih.gov/categorical_spending.aspx.

*Source:* Official federal agency budgets.

Turning from the total funding estimates for health services research using agency reported publicly available data, HSRProj enables comparison of the number of projects (as opposed to their dollar amount) by funding agency (HSRProj). Looking across all public and private sector sources of health services research support between 2005 and 2016, NIH has consistently funded the largest number of projects (between 43 percent and 56 percent). In 2016, AHRQ funded the second highest number of projects (245) followed by PCORI and the Department of Veterans Affairs (VA) (Table 2). Looking only at the top eight funders listed in Table 2, there has been a 20 percent decline in the number of projects over the 11‐year period. This may reflect a real overall decrease in support or a trend toward a smaller number of larger funded projects. There was a noticeable increase in the number of projects at NIH and the VA in 2009 after the infusion of $1.1 billion to support comparative effectiveness research (CER), but this increase was not sustained. Of note is the sharp decrease in the number of projects supported by the Robert Wood Johnson Foundation after the 2008 recession, a trend that continued as the Foundation's vision shifted to broader concerns of a culture of health resulting in the elimination of numerous long‐standing programs.

**Table 2:** Number of projects supported by top health services research funders, 2005–2016

	2005	2007	2009	2011	2013	2015	2016	Change (%) between 2005 and 2016
National Institutes of Health (combined)	630	561	845	619	630	513	586	−6.98
Robert Wood Johnson Foundation (RWJF)	339	294	189	138	130	68	51	−84.9
Agency for Healthcare Research and Quality (AHRQ)	122	186	206	140	243	232	245	100.8
Centers for Medicare and Medicaid Services (CMS)	95	31	22	11	13	8	12	−87.4
Health Resources and Services Administration (HRSA), Office of Rural Health Policy	81	22	27	20	28	27	8	−90.1
Department of Veterans Affairs (VA)	68	101	187	167	178	105	87	27.9
Patient‐Centered Outcomes Research Institute (PCORI)	–	–	–	–	136	117	117	−14.0 (change 2013 and 16)
Commonwealth Fund	50	100	42	112	38	61	–	26.0 (change 2005 and 15)
Total	1385	1295	1518	1207	1396	1131	1106	−20.14

*Source:* HSRProj, 2017.

Turning to NIH‐supported health services research across the Institutes and Centers, there has been an overall 7 percent decrease in the number of projects supported between 2005 and 2016 (Table 2). The largest reductions were at the National Institute of Mental Health and National Cancer Institute (65 percent and 46 percent, respectively; Table 3), followed by the National Institute of Drug Abuse (29 percent) and the National Institute for Child Health and Human Development (18 percent). In 2016, the National Institute of Mental Health and the National Institute on Aging supported the greatest number of health services research projects (66 projects each). The concentration of health services research projects funded by the top funding NIH institutes decreased across the decade represented in these data.

**Table 3:** Number of Health Services Research Projects Supported by NIH Institutes, 2005–2016

Year:	2005	2007	2009	2011	2013	2015	2016	Change (%) between 2005 and 2016
National Institute of Mental Health (NIMH)	191	116	122	68	63	52	66	−65.1
National Cancer Institute (NCI)	91	89	155	104	58	46	49	−46.2
National Institute on Drug Abuse (NIDA)	58	64	77	51	55	46	41	−29.3
National Institute of Child Health and Human Development (NICHD)	51	56	73	58	68	38	42	−17.6
National Institute of Nursing Research (NINR)	41	20	35	39	37	15	35	−14.6
National Institute on Aging (NIA)	38	46	55	46	67	60	66	−73.7
National Institute on Minority Health and Health Disparities (NIMHD)	39	22	49	38	37	18	51	30.8
National Heart, Lung, and Blood Institute (NHLBI)	32	38	70	41	32	24	51	59.4
National Institute of Diabetes and Digestive and Kidney Diseases (NIDDK)	22	20	33	49	22	42	30	36.4
National Institute on Alcohol Abuse and Alcoholism (NIAAA)	21	21	24	22	17	13	17	−19.0
National Institute of Dental and Craniofacial Research (NIDCR)	7	11	13	22	13	23	12	71.4
National Institute of Allergy and Infectious Diseases (NIAID)	6	1	7	3	5	6	5	−16.7
Fogarty International Center (FIC)	7	8	12	14	21	12	14	100.0
National Institute of Arthritis and Musculoskeletal and Skin Diseases (NIAMS)	5	12	16	12	7	3	–	−40.0 (change between 2005 and 2015)
National Institute of Neurological Disorders and Stroke (NINDS)	5	10	9	11	23	3	2	−60.0
National Center for Complementary and Integrative Health (NCCIH)	5	2	17	12	12	6	15	275.0
National Library of Medicine (NLM)	4	0	7	0	7	12	15	275.0
National Center for Research Resources (NCRR)	2	5	12	2	–	–	–	0 (change between 2005 and 2011. Became NCATS.)
National Human Genome Research Institute (NHGRI)	2	2	5	11	13	20	11	450.0
National Eye Institute (NEI)	1	3	9	3	9	12	7	600.0
National Institute of General Medical Sciences (NIGMS)	1	0	5	0	7	5	2	100.0
National Center for Advancing Translational Sciences (NCATS)	–	11	9	5	38	34	25	127.2 (change between 2007 and 2016)
National Institute on Deafness and Other Communication Disorders (NIDCD)	0	2	3	4	8	9	8	–
Office of the Director (OD)	1	1	24	4	6	6	17	1,600.0
National Institute of Biomedical Imaging and Bioengineering (NIBIB)	0	1	3	0	3	4	5	–
National Institute of Environmental Health Sciences (NIEHS)	0	0	1	0	2	4	–	–
National Institutes of Health (total)	630	561	845	619	630	513	586	−6.98

*Source:* HSRProj, 2017.

In a reversal of these trends of reductions and/or stagnation in federal support for health services research, the final FY 2018 budget provided significant increases for research funding across multiple agencies, including a $3 billion more for NIH, an 8.3 percent increase to $37 billion (Science Magazine). Of note, in this budget deal, AHRQ received a small increase—$10 million—but one that is notable as the first increase in 9 years. However, it is not clear at this time whether the FY 2018 increases are auguring in a new trend of enhanced support for health services research. In fact, the FY 2019 proposed budget by the Trump administration continues to include proposed cuts to AHRQ and other HHS entities and for the second time eliminates AHRQ and creates instead a National Institute for Research on Safety and Quality, a structural change that raises numerous questions for all health services research stakeholders.

What the above data do not shed light on is the degree to which the private sector is supporting health services research. A recent study estimated overall funding for health services research to be between 0.2 and 0.3 percent of national health care expenditures between 2003 and 2011 (Moses et al. 2015). This higher estimates stem from the fact that the authors estimated total health services research funding at $5 billion in 2011, an estimate significantly higher because of two factors. First, 2011 was a year that AHRQ's budget was enhanced by the one‐time investment in CER, and second, their total health services research funding includes $1.4 billion in estimated health services research funding from the health services industry, including hospitals and other health care provider organizations. Moses et al. noted that while the estimate of health industry support may be an underestimate, it is still very low compared to other industrial sectors. Health services companies invest just 0.1 percent of revenue in health services research compared to 1.7–2.5 percent of revenue invested in research and development in other sectors of the economy.

## Conclusion

The future size, scope, and focus of federal support for health services research remain uncertain given recent trends including continued pressures on federal discretionary spending. While the administration's proposal to move AHRQ into the NIH as part of the FY 2018 budget and again in FY 2019 was roundly rejected by Congress, the Congress did include in the Agency's budget a requirement to conduct a study to “identify research gaps and areas for consolidation, as well as propose strategies for better coordination of the Federal health services research enterprise,” signaling their willingness to consider other structural options for funding and coordination of health services research. This could prove to be an opportunity for the health services research community to step back and assess the changing nature, purpose and impact of health services research, and the priorities for federal support, including support for research, data, and training.

## Supporting information

Appendix SA1: Author Matrix.

## Note

1 This is the latest year with data available for the Patient Centered Outcomes Research Institute (PCORI).

## References

[hesr13040-bib-0001] Centers for Medicare and Medicaid Services, National Health Expenditures Fact Sheet . 2018 Available at https://www.cms.gov/research-statistics-data-and-systems/statistics-trends-and-reports/nationalhealthexpenddata/nhe-fact-sheet.html [accessed on March 4, 2018].

[hesr13040-bib-0002] HSRProj . 2017 HSRProj Research Includes Studies and Papers on a Variety of Topics. Inclusion and exclusion criteria can be found here: http://www.academyhealth.org/hsrprojsubmit. Interactive charts are available here: http://www.academyhealth.org/hsrprojcharts [accessed on April 22, 2018].

[hesr13040-bib-0003] Moses, H. , D. H. Matheson , S. Cairns‐Smith , B. P. George , C. Palisch , and E. R. Dorsey . 2015 “The Anatomy of Medical Research: US and International Comparisons.” Journal of the American Medical Association 313 (2): 174–89. 25585329 10.1001/jama.2014.15939

[hesr13040-bib-0004] NIH Reporter . Available at https://report.nih.gov/categorical_spending.aspx [accessed on July 26, 2018].

[hesr13040-bib-0005] Science Magazine . “Trump, Congress Approve Largest U.S. Research Spending Increase in a Decade” [accessed on March 26, 2018]. Available at http://www.sciencemag.org/news/2018/03/updated-us-spending-deal-contains-largest-research-spending-increase-decade

